# Automated quantitative analysis of Ki-67 staining and HE images recognition and registration based on whole tissue sections in breast carcinoma

**DOI:** 10.1186/s13000-020-00957-5

**Published:** 2020-05-29

**Authors:** Min Feng, Yang Deng, Libo Yang, Qiuyang Jing, Zhang Zhang, Lian Xu, Xiaoxia Wei, Yanyan Zhou, Diwei Wu, Fei Xiang, Yizhe Wang, Ji Bao, Hong Bu

**Affiliations:** 1 Laboratory of Pathology, West China Hospital, Sichuan University, Chengdu, 610041 China; 2 Department of Pathology, West China Second University Hospital, Sichuan University & key Laboratory of Birth Defects and Related Diseases of Women and Children (Sichuan University), Ministry of Education, Chengdu, 610041 China; 3 Department of Pathology, West China Hospital, Sichuan University, Chengdu, 610041 China; 4 Department of Pathology, Chengfei Hospital, Chengdu, China; 5 Chengdu Knowledge Vision Science and Technology Co., Ltd, Chengdu, China

**Keywords:** Convolutional neural network, Whole tissue sections, Breast invasive ductal carcinoma, Automatic recognition, Ki-67 counting

## Abstract

**Background:**

The scoring of Ki-67 is highly relevant for the diagnosis, classification, prognosis, and treatment in breast invasive ductal carcinoma (IDC). Traditional scoring method of Ki-67 staining followed by manual counting, is time-consumption and inter−/intra observer variability, which may limit its clinical value. Although more and more algorithms and individual platforms have been developed for the assessment of Ki-67 stained images to improve its accuracy level, most of them lack of accurate registration of immunohistochemical (IHC) images and their matched hematoxylin-eosin (HE) images, or did not accurately labelled each positive and negative cell with Ki-67 staining based on whole tissue sections (WTS). In view of this, we introduce an accurate image registration method and an automatic identification and counting software of Ki-67 based on WTS by deep learning.

**Methods:**

We marked 1017 breast IDC whole slide imaging (WSI), established a research workflow based on the (i) identification of IDC area, (ii) registration of HE and IHC slides from the same anatomical region, and (iii) counting of positive Ki-67 staining.

**Results:**

The accuracy, sensitivity, and specificity levels of identifying breast IDC regions were 89.44, 85.05, and 95.23%, respectively, and the contiguous HE and Ki-67 stained slides perfectly registered. We counted and labelled each cell of 10 Ki-67 slides as standard for testing on WTS, the accuracy by automatic calculation of Ki-67 positive rate in attained IDC was 90.2%. In the human-machine competition of Ki-67 scoring, the average time of 1 slide was 2.3 min with 1 GPU by using this software, and the accuracy was 99.4%, which was over 90% of the results provided by participating doctors.

**Conclusions:**

Our study demonstrates the enormous potential of automated quantitative analysis of Ki-67 staining and HE images recognition and registration based on WTS, and the automated scoring of Ki67 can thus successfully address issues of consistency, reproducibility and accuracy. We will provide those labelled images as an open-free platform for researchers to assess the performance of computer algorithms for automated Ki-67 scoring on IHC stained slides.

## Introduction

Breast invasive ductal carcinoma (IDC) is the most common malignant tumor in women worldwide, with a trend of younger at diagnosis [1, 2]. In 2018, there were more than 266,000 new cases of breast cancer in women in the United States, accounting for 30% of all malignant tumors in women and far exceeding the second lung cancer (13%) [3]. In both developed and developing countries, the disease ranks as third in the mortality rate among females [2, 3]. Ki-67 protein, as well as ER, PR, and HER-2 protein, have been recognized as main biological indicators to guide the molecular typing, treatment plan, and prognosis evaluation of breast cancer [4]. Ki-67 is a cell cycle related nucleoprotein, which has been served as an accurate marker to infer the proliferative status of tumor cells, since it only reacts with the proliferating cells and shows no tissue specificity [5]. Interestingly, a number of studies have reported that Ki-67 staining can be used as a reference index for the prognosis and personalized treatment of breast cancer patients, it is also closely related to the clinicopathological features and molecular typing of breast cancer patients [5–7]. Moreover, Ki-67 scoring can be used to distinguish luminal breast cancer subtypes (A/B) and, as a result, it certainly helps to define the best treatment strategy for each particular condition [8, 9]. In triple negative breast cancer (TNBC), patients high Ki-67 scores seem to benefit more from the treatment [10].

Nevertheless, the traditional scoring method of Ki-67 staining by IHC, can be frequently time-consuming, labor-intensive, and poorly reproducible for many pathologists, and later provide limited reproducibility and quantification of respective markers. These common problems can seriously hinder the establishment and management of patient treatment, especially during late phases. Fortunately, with the emergence of whole slide digital scanning technology, it is now feasible to combine histopathological image information with artificial intelligence (AI) technology. This combination meets the standards of high definition, high speed, and high throughput screening [11], which could lay a good foundation for the development and application of digital pathology. Using whole slide imaging (WSI) as the basis, combined with a series of technical equipment including (i) a image analysis system and (ii) an information management system, via deep learning of the computer, AI can effectively simulate a pathologist’s brain for effective thinking and further assist in broader applications in the medical and health areas, such as disease intelligence analysis, tumour-assisted diagnosis, gene data detection, and disease drug development [12–15]. Generally, WSIs are gigapixel images stored in a multi-resolution pyramid structure where the highest resolution is × 40. Moreover, a model training based on convolutional neural networks (CNN) may provide doctors with effective and accurate information, such as pathological disease typing, cancer histology-assisted diagnosis, mitotic cell counts, epithelium–stroma classification, lymph node metastasis assessment and others [16–21]. CNN techniques are guided by structural and statistical information derived from respective images. There are several deep learning models described so far, in particular for CNN, such as LeNet, AlexNet, and GoogleNet [22]. Hence, the question arises whether AI could be used to solve the problem of accurate counting of Ki-67 on immunohistochemically stained sections. Existing research has revealed that the development of counting softwares, focusing on Ki-67 staining in a variety of tumors, still have many limitations, including the lack of automated location for areas of interest, or accurate registration of IHC images and their HE images. To attempt providing stronger assessment, reliable comparisons, and more reproducible results, here we utilized simulated data to compare analytical performance among different algorithms, and we further selected an unsupervised domain adaptation for counting, based on few simple and easily-implemented CNN models, named as GoogLeNet Inception V1, this model could help us located the IDC area automatically. And then, we registered the labelled HE and Ki-67 stained sections using a Simple Elastix toolbox, which was developed by our engineer teams to handle medical image registration issues specifically. Finally, we used an algorithm provided by Image J to automatically extract the structure, morphology, color, and other characteristics of positive/negative cells, and train the random forest classifier that could identify Ki-67 positive/negative cells. In addition, we marked 10 standard Ki-67 Counting slides for testing on whole tissue sections, these slides were labelled by ten pathologists, who circled each cell in the tumor region of these slides and determined whether it was positive or negative.

## Materials and methods

### Experimental design

**Fig. 1:**
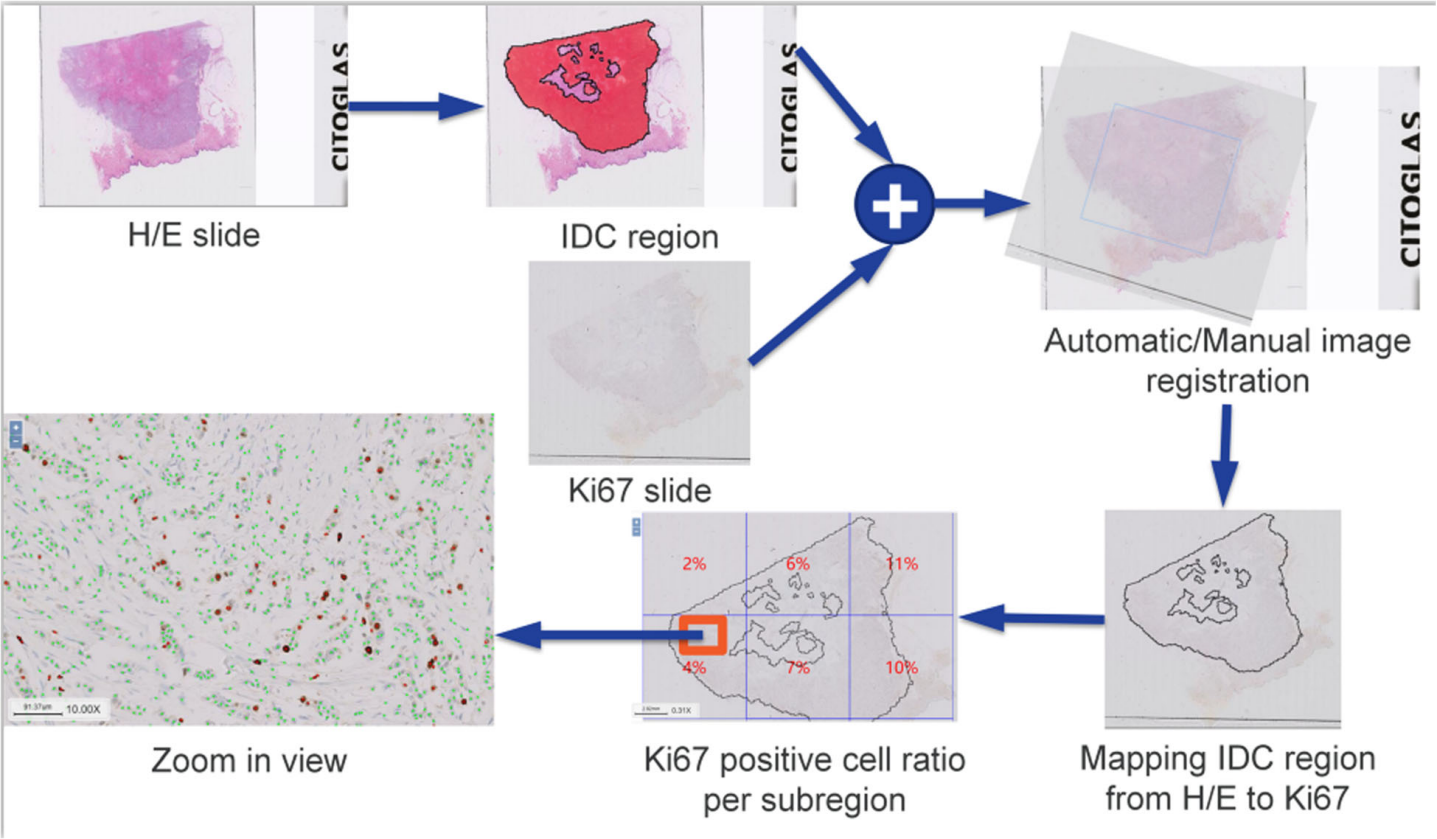
The flow chart of Ki-67 Automatic Counting Software in breast IDC on whole tissue sections

Research process was divided into three stages: identification of IDC, registration, and enumerating of Ki-67 staining (See Fig. 1 for the flow chart of Ki-67 Automatic Counting Software in breast IDC on WSI). To enhance the classification performance of IDC and ductal carcinoma in situ (DCIS), and, simultaneously, to reduce the network training time, our method was designed with unsupervised domain adaptation for counting, based on GoogLeNet Inception V1.

### Case selection

A total of 1074 IDC slides from 672 cases diagnosed by the Department of Pathology of West China Hospital (Sichuan University, China) were collected. From these, 57 unqualified sections were removed after primary screening due to (i) quality issues with the sections, (ii) insufficient scanning clarity, and/or (iii) poor identification of IDC area. The remaining 1017 sections were processed for this study at last. We randomly selected 677 of these sections as training sets, 153 as verification sets, and 187 as test sets. Both H&E and Ki-67 stained slides were collated into a complete digital scanning section WSI by digital section scanner (Hamamatsu Optics’ NanoZoomer 2.0HT), with a magnification of 40 × .

### Immunohistochemistry

For immunohistochemical staining of Ki-67, 4 um thin tissue sections were dewaxed in xylene, acetone and Tris-buffered saline, followed by heat induced epitope retrieval in pH 6.0 in a microwave oven (750 W). Ventana was used for antigen retrieval. Sections were subsequently stained using Ki67 antibody (clone mAb, ready-to-use formulation), purchasing from Roche. All the steps were carried out according to the instructions and stained by Bench Mark ULTRA automatic immunohistochemical staining machine.

### Image acquisition

At this stage, we have included 1017 breast IDC diagnosed slides marked with the IDC regions, followed by the removal of features related to these labelled digital slides. The classification network model was further trained by GoogleNet Concept V1, which could be used to automatically identify IDC regions.

### Labelling

A team of 36 pathologists from West China Clinical Medical College (Sichuan University, China) was organized in order to label the IDC area on the WSI. Both workflow and number of pathologists involved were divided into four categories: (i) WSI labelling (28 professionals), (ii) labelling review (3 professionals), (iii) labelling quality control (2 professionals), and (iv) training experts (3 professionals). Firstly, three experts from the Breast Diseases Group, Chinese Medical Association Pathology Branch, conducted multiple training sessions to appropriately distinguish the IDC regions in the WSI. Next, 28 labelling staff members were divided into other three groups to complete the labelling of IDC regions in all WSI. Simultaneously, a pathologist with intermediate or above titles was assigned as the team leader for each group, to review the labelled regions and to provide feedback on the results to the labelling staff in a timely manner. Meanwhile, two attending pathologists were appointed as quality control physicians to conduct random checks on WSI after reviewing (random rate of 5% or above), with an accuracy rate of more than 95% for proper qualification. For labelling, we used different colors to distinguish various tissue regions.

### Training

After labelling by the pathologists, software engineer used the computer image processing algorithm to segment and extract the labelled information, classifying and extracting the positive and negative regions accordingly (128 pixel × 128 pixel patch), and then used GoogleNet Incubation V1 for featured extraction and classification training to obtain a network model. At this stage, 677 training sets of WSI were used to fit the parameters of the model, while 153 verification sets of WSI were used to tune the model hyperparameters during training procedures (Table 1). A total of 2000 positive patches and 2000 negative patches were selected for training in each WSI, whereas the redundant patches were not included in the training set.

**Table 1:** The results of segmentation and extraction based on 1017 HE slices labelled information

Type of sets	training sets	verification sets	test sets	Sum
WSI number	677	153	187	1017
Patch number	11,628,208	2,973,384	2,419,032	17,020,624

### Testing

After training the classification model, 187 test sets’ of WSIs were used to provide an unbiased evaluation of a final model fit on the training dataset. We took each patch as a unit, and then considered the IDC area pre-marked by pathologists as a “gold standard”. Next, we compared it with the analytical results of AI systems to retrieve performance indicators such as sensitivity, specificity, and accuracy. Labelling was strictly confidential before testing, to meet the requirements of “a single-blind” study.

### Registration

We randomly selected 100 cases with both HE slides and their corresponding Ki-67 stained slides, which were created by serial sectioning technique. Next, we registered the labelled HE and Ki-67 stained sections using a Simple Elastix toolbox, which developed by our engineer teams, could handle medical image registration issues. Slides were initially superimposed by this toolbox, and then automatically modified into a rigid transformation such as translation and rotation via the registration function of the tool, thereby achieving a good registration effect. Eventually, labelling of each HE slides was migrated to respective Ki-67 images, and the IDC area on each Ki-67 slice was selected accordingly.

### Counting of Ki-67 stained sections on whole tissue section

The registered Ki-67 stained sections were labelled to identify the positive and negative tumour cells. According to the labelling information, we used an algorithm provided by Image J (an open source software for digital pathology image analysis) to automatically extract the structure, morphology, colour, and other characteristics of positive/negative cells, and train the random forest classifier that could identify Ki-67 positive/negative cells. This procedure allowed the automatic counting of Ki-67 positive and negative cells in the IDC region and, as required, Ki-67 positive rate. At this stage, we circled ten ROI (region of interest) on each Ki-67 slice, where each ROI included at least 100 cells. More than 100,000 cells in all were labelled, in which positive and negative cells were marked, respectively, in red and blue colours. The Ki-67 positive rate calculated from these artificially labelled cells is considered to be the “gold standard”. These labelled cells were also used to tune the Ki-67 counting model at the verification sets.

### Testing of the total accuracy

After the aforementioned stages, we acquired an integrated WSI-based model for Ki-67 Automatic Counting in breast invasive ductal carcinoma. Thereafter, we tested the accuracy rate of this Ki-67 counting model. In addition, we organized a competition test, featuring ten clinical pathologists, to verify the modelling efficiency.

### Labelling of standard Ki-67 counting

Ten HE and Ki-67 co-stained IDC sections (excluding intraductal carcinoma tissues), originated from different patients, were used as standard provided by the Department of Pathology of the Sichuan Cancer Hospital. These sections were labelled by ten pathologists of West China Hospital (Sichuan University, China). Pathological staff also participated in the labelling of Ki-67 automatic analysis system in our IDC study by determining the number and positive rate of Ki-67 staining in respective areas. Results were classified as standard for this testing.

### Testing and competition

The Ki-67 Artificial Intelligence Counting System developed by our institute was presented at the “2017 Pathological Image Diagnosis Human-Machine Challenge” (seventh China Pathology Annual Meeting). Contestants competed with ten senior pathologists to validate the modelling efficiency. Competition was based on the independent completion of Ki-67 positive counting in IDC areas of ten breast cancer WSI within 30 min. For this, results were required to be accurate to 1%; the completion time of each contestant was recorded by auxiliary personnel. Completion time and accuracy of each contestant were comprehensively evaluated.

## Results

### IDC identification testing

**Fig. 2:**
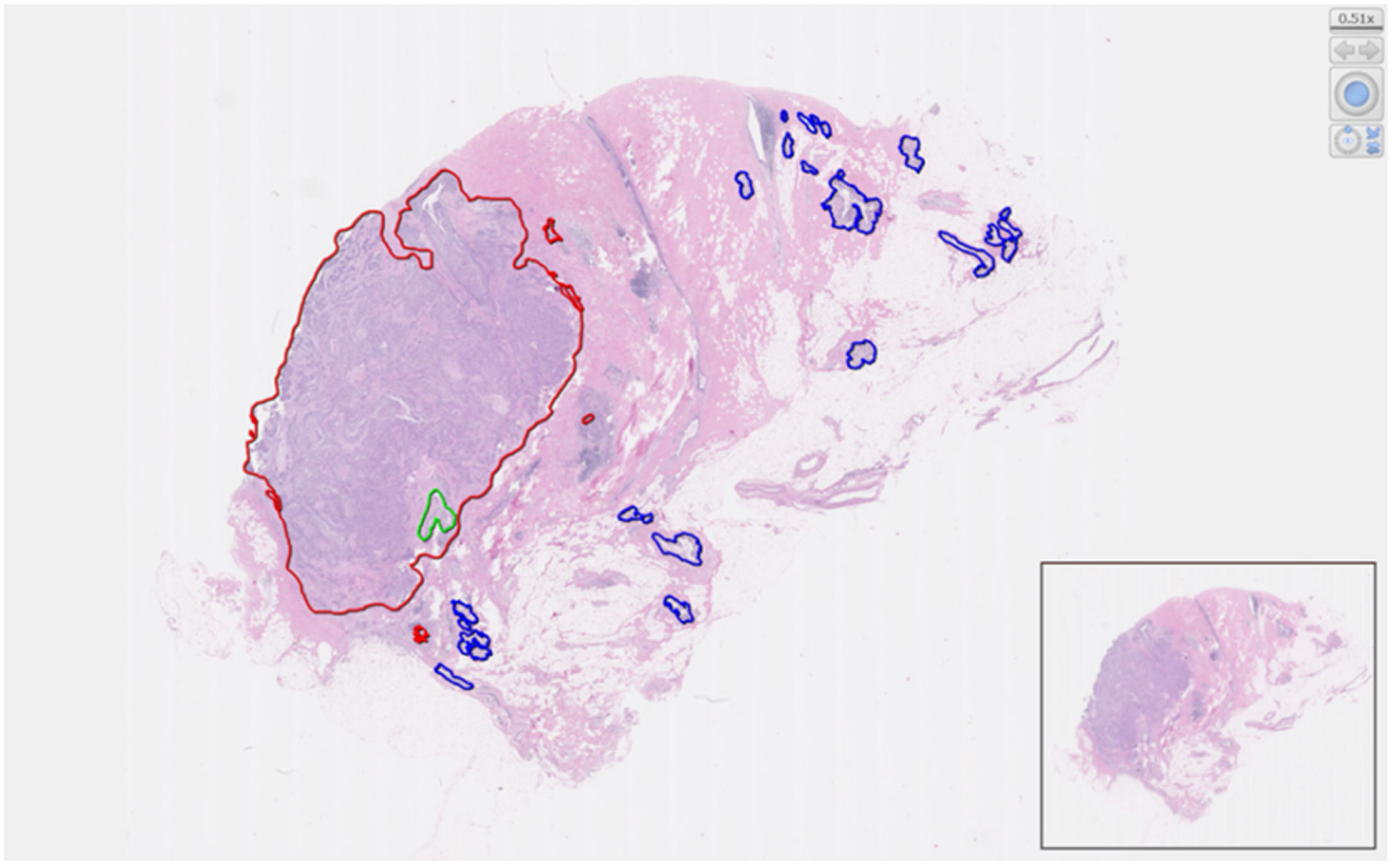
Comparative pathological analysis of breast tissue regions. Regions related to breast IDC (red), ductal carcinoma in situ (DCIS) (green), and normal breast tissue (blue) are shown

**Fig. 3:**
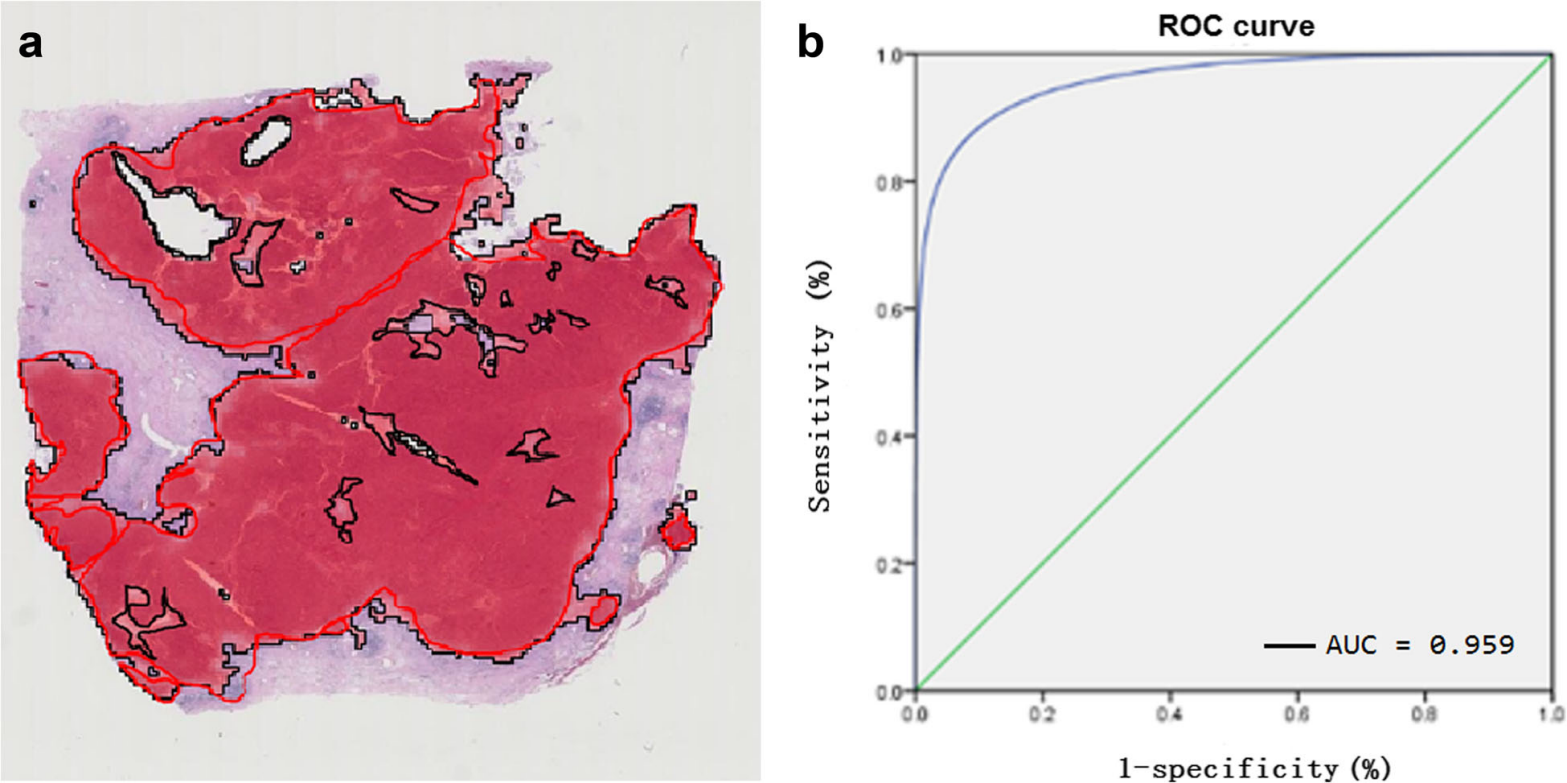
Comparison of the test system and the standard. **a**, Black box with red fields indicates the heat map, which was obtained by GoogLeNet Inception V1. Red lasso region relates to the breast IDC region marked by the pathology team (considered it as “gold standard”). **b**, ROC curve of the breast IDC identification based on WSI, the area under curve is 0.959

Before training, we segmented 1017 WSIs into (i) 677 training sets, (ii) 153 verification sets, and (iii) 187 test sets (Table 1). We used different colors to distinguish IDC and DCIS, and normal breast tissue regions for further labelling on each WSI (Fig. 2). When testing this research system, we created a heatmap by calculating and comparing it with the “gold standard”, which was properly defined by the pathologists (Fig. 3a). In addition, a “blind method” design was adopted for this test. The final test results indicated that the sensitivity of computer automatic identification of IDC region was 85.05% (misdiagnosis rate of 14.95%), specificity was 95.23% (misdiagnosis rate of 4.77%), accuracy was 89.44%, balance accuracy was 90.14%, and AUC value was 0.959 (Table 2, Fig. 3b).

**Table 2:** Test Results of breast IDC identification based on whole slide imaging

Test Indicators	Test Results
Sensitivity	0.8505
Specificity	0.9523
Balance accuracy	0.9014
Accuracy	0.8944
Positive result likehood ratio (PRLR)	17.84
Negative result likehood ratio (LR)	0.16
Positive predictive values	0.9592
Negative predictive values	0.8286
Diagnostic index	1.8028
Youden index	0.8028
False positive rate	0.0477
False negative rate	0.1495

### Registration results of Ki-67 staining and corresponding IDC region

**Fig. 4:**
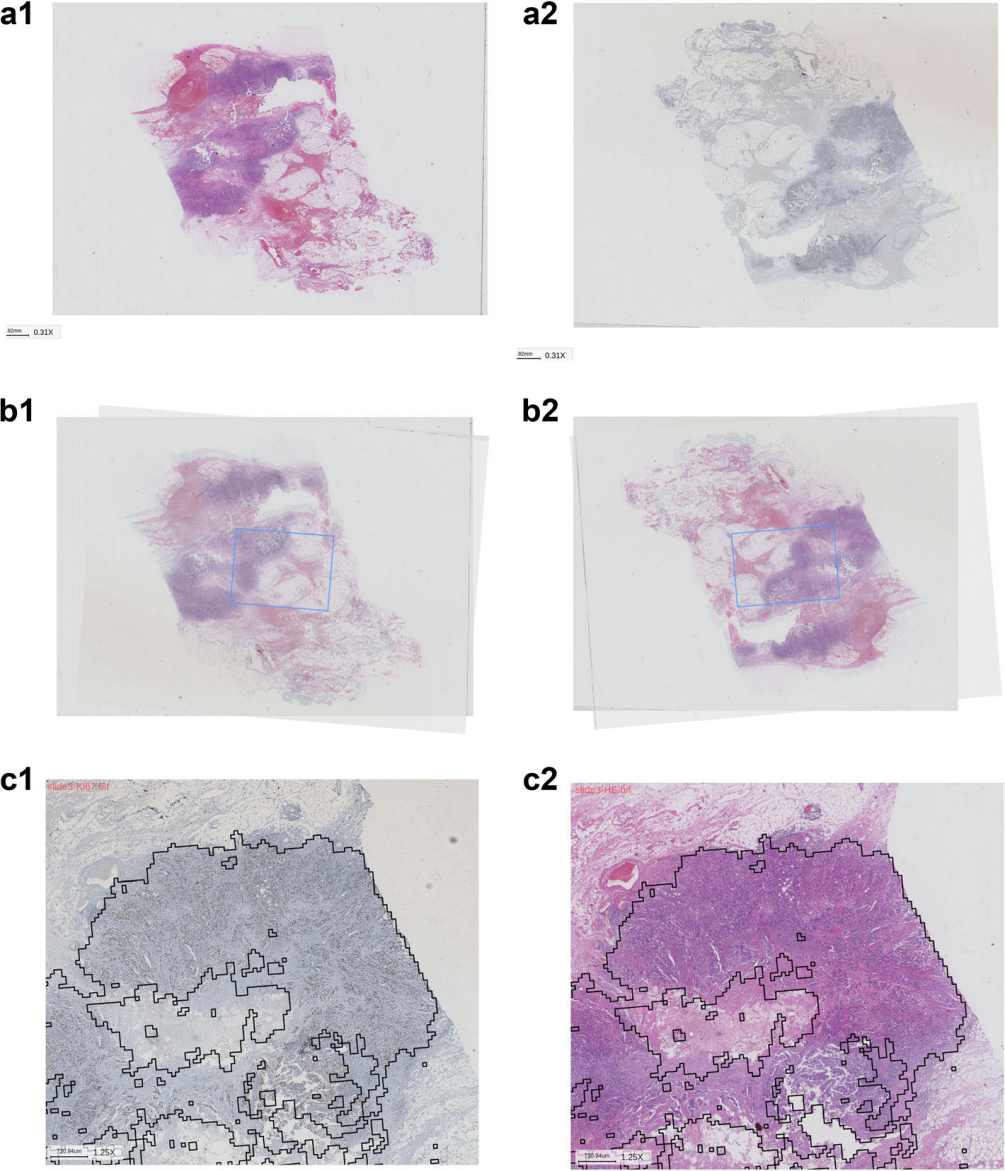
Ki-67 staining and corresponding registration results of IDC regions. The figure illustrates contiguous HE slides and Ki-67 stained slides that were perfectly registered (in most cases). **a**, Contiguous HE slides and Ki-67 stained slides. **b**, Registering. **c**, Registration results of IDC region in the Ki-67 slides

We selected 100 cases with both HE and Ki-67 stained slides, which were created by a serial sectioning technique for registration. Results revealed that contiguous HE and Ki-67 stained slides could be perfectly registered, and considerably fewer HE and Ki-67 stained slides, with larger differences, could register the core areas (Fig. 4a-c).

### Ki-67 positive rate test results

Simultaneously, we tested the Ki-67 positive rate in the IDC area. The results revealed that the accuracy of Ki-67 positive rate in the IDC could attain 90.2% after only a few minutes of automatic calculation, using the algorithm provided by Image J. An additional movie file shows this in more detail (see Additional file 1).

### Manual labelling of gold standard results for Ki-67 positive cells

**Fig. 5:**
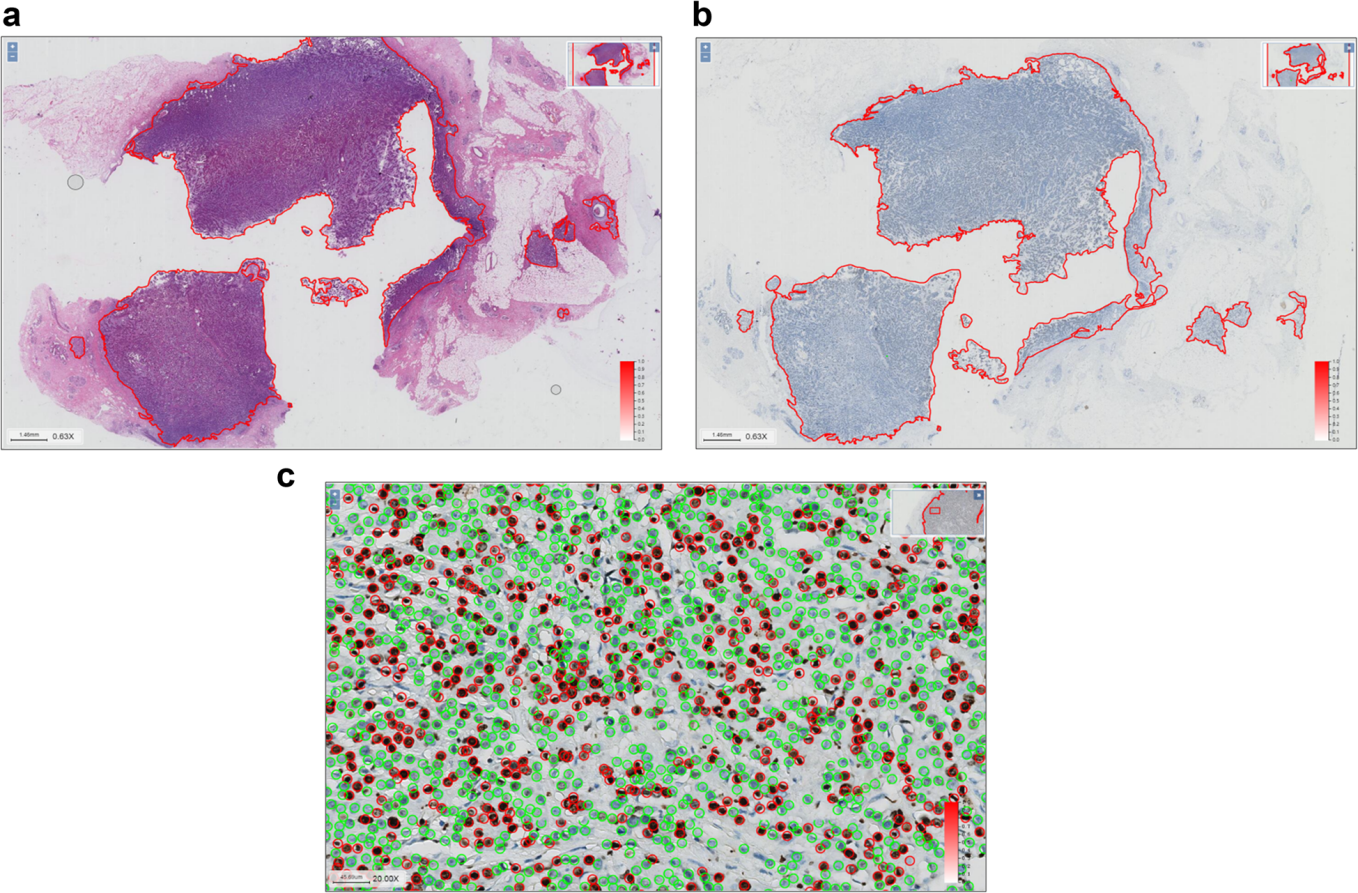
Manual labelling of “gold standard” results for Ki-67 positive cells. **a**, Selected regions of breast IDC on HE slides. **b**, Corresponding regions of breast IDC on Ki-67 stained slides. **c**, Tumour cells in IDC regions on Ki-67 stained slides (red for positive cells, green for negative cells)

During the human–machine challenge, our labelling staff manually labelled the Ki-67 positive and negative tumor cells in the IDC area of the WSI, with an average of more than 200,000 cells per person and over 80 h of intensive work, thereby providing the most accurate Ki-67 index score to date (Fig. 5a-c, Table 3).

**Table 3:** Manual labelling of “gold standard” results for Ki-67 positive cells in the human-machine challenge

number	Positive nuclei count	Negative nuclei count	Total number of cells	Ki-67 Index score (%)	Standard score
1	29,961	299,428	329,389	9.10	9 or 10
2	50,073	270,593	320,666	15.62	15 or 16
3	31,119	73,719	104,838	29.68	29 or 30
4	20,272	109,013	129,285	15.68	15 or 16
5	9854	79,026	88,880	11.09	11 or 12
6	11,939	122,641	134,580	8.87	8 or 9
7	9332	100,608	109,940	8.49	8 or 9
8	232,515	86,582	319,097	72.87	72 or 73
9	30,003	270,266	300,266	9.99	9 or 10
10	85,036	69,088	154,124	55.17	55 or 56
Sum	510,104	1,480,964	1,991,068	–	
Average	51,010	148,096	199,107	25.62	

### Competition results

The final results of this Human-Machine Competition showed that the automatic counting system we developed had an accuracy rate of 99.4% in this challenge, which was over 90% of the results provided by participating doctors. The time that AI spent for 10 slides was 23′19″, which was less than the average time of manual counting 25′31 s″. The AI system lagged behind the pathologist from Hebei Medical University by 0.1 points and won second place (see Table 4 for the competition results of all contestants).

**Table 4:** Details for the competition results of all contestants

No.	Dr. NO.1 score	Dr. NO.2 score	Dr. NO.3 score	Dr. NO.4 score	Dr. NO.5 score	Dr. NO.6 score	Dr. NO.7 score	Dr. NO.8 score	Dr. NO.9 score	Dr.NO.10 score	Average score	AI score
1	94	84	91	99	94	96	100	99	92	80	92.9	100
2	96	86	91	89	94	95	99	94	96	95	93.5	98
3	90	98	90	85	84	69	100	65	70	88	83.9	97
4	98	92	92	88	69	89	99	99	74	99	89.9	99
5	99	91	86	92	97	90	98	97	98	93	94.1	99
6	96	93	96	92	96	99	99	97	96	97	96.1	100
7	86	93	52	97	68	88	98	100	88	78	84.8	99
8	92	98	98	78	88	88	100	78	83	98	90.1	98
9	97	100	95	96	92	96	98	95	90	90	93.9	99
10	100	70	70	75	65	93	99	65	90	68	79.5	100
Average score	94.8	90.5	86.1	89.1	84.7	89.3	99.0	88.9	87.7	88.6		98.9
Total time	28′27″	25′54″	28′56″	28′25″	20′33″	23′40″	18′46″	20′23″	26′55″	27′42″	25′31″	23′19″

## Discussion

Due to the continuous increase on the incidence of breast cancer worldwide, especially at younger ages, more focus has been dedicated to the treatment and prognosis of this malignancy. Ki-67 is a well-established biomarker closely related to the development, metastasis, and prognosis of various tumors. In fact, Ki-67 is considered one of the most important protein markers to be evaluated in clinicopathological applications in breast cancer [1, 12]. So far, several researches reveal that Ki-67 automatic counting systems and individual platforms, such as Immuno Path and Immuno Ratio softwares, have been developed and further utilized in lung cancer, pancreatic cancer, lymphoma, breast cancer, and other tumors [23, 24]. Still, most of these systems could not meet the need of automation in clinical medicine, since the existing Ki-67 algorithms cannot automatically find the focused tissue regions, or automatically complete registration of IHC images and their HE images. Our work embraces the field of image recognition and registration, and applies a model of classification based on convolution network, using AI for the automatic identification of IDC regions and combining it with the traditional computer based Ki-67 positive algorithms. Therefore, this combination not only allowed the development of an effective method to extract the image ridge feature for Ki-67-stained IHC images and their HE images accurate registration automatically in breast IDC based on whole tissue sections, and obtained good results, but also developed a Ki-67 automatic counting software based on previous accurate image registration. Our results indicate that this new technological approach is feasible, efficient, and accurate for IHC images and their HE images registration and automatic scoring of Ki-67. What’s more, we provide those accurately labeled digital images of each positive and negative cells of ki-67 staining as an free-open public platform for researchers to assess the performance of computer algorithms for automated Ki-67 scoring on IHC stained slides.

WSI-based digital pathology has revealed immense advantages over traditional pathology diagnosis mode [3]. Several domestic and foreign pathology teaching and research departments have already used WSI for hardware conditions on daily pathological diagnosis and scientific research experiments [23–25]. The accurate and efficient labelling of the targeted WSI area is the key to digital pathology-related research [25]. In fact, the key first step of this study was to appropriately label the IDC regions in WSI images to provide computers with reliable and accurate data information learning. Through this study, we have explored a set of feasible programs and procedures for training labelling personnel based on WSI images, and, moreover, we have strengthened the role of pathologists in computer-aided diagnosis and analysis.

At present, the most commonly used evaluation method of registration effect is based on gray level, just like sum square differences (SSD), Normalized Mutual Information (NMI) and normalized cross correlation (NCC). In this paper, we choose NCC as our evaluation method of registration. It calculates the matching degree between two graphs by normalized correlation measurement formula. NCC evaluation algorithm can effectively reduce the impact of light on image comparison results, and the results of NCC evaluation algorithm are normalized to between 0 and 1, which is easy to quantify and judge the quality of registration results. The NCC value of our registration model is 0.975, this shows that the matching degree is very good and sufficient to meet the actual needs. In addition, automatic registration should produced some areas that do not match perfectly, for these areas, we had tried to manually adjust them to match perfectly. However, the test result found that the difference of the positive rate of IHC sections between manually adjusted and automatic results were very small. Our analysis suggested that was because the registration model had been able to make the WSIs highly matched, and slight regional differences in registration had little impact on the final result.

While performing slide screening and classification model training, it was necessary to continuously interpolate the verified experiments in order to improve the training efficiency and accuracy of the classification model. We found that a few non-standard pathological sections (such as IDC areas not appropriate for identification, and positive areas of unexpected dimensions) could reduce the accuracy of the classification model. The main reason appeared to be that the accuracy of the classifier was affected by differences in the individual characteristics of the image, possibly greater than the differences in the classification characteristics. For instance, when the number of patches extracted from a WSI was particularly large or small, the features learned by computer classification model may not represent the expected classification characteristics (such as IDC’s image characteristics) but, instead, they might be peculiar to the individual image that was evaluated (such as color differences and/or impurities of the present image). A potential alternative was prepared by selecting per WSI for training (2 k positive and 2 k negative patches were selected in our study), whereas the redundant patches were not included in the training set. Therefore, while selecting slides, we had to select proper types with obvious IDC area and moderate size, which would be more conducive to retrieve an accurate classification model. This revealed that a verification step was essential, and it required constant exchange of experience between the pathology team and the computer engineer team, as well as a close cooperation between these groups for troubleshooting purposes.

Internationally, automatic analysis with the aid of artificial intelligence has covered a variety of diseases, ranging from “benign” conditions such as diabetic retinopathy and Alzheimer’s disease [7], to malignant tumors such as breast cancer [26–28], lung cancer [29], liver cancer [30], skin cancer [31], osteosarcoma [32], and lymphoma [33, 34], with an accuracy rate of 89.4–97.8%, and an AUC score of 0.85–0.94 [7, 27, 31]. In addition, various AI systems related to breast cancer have penetrated through different levels of IDC, such as histology-assisted and cytology-assisted diagnosis, mitotic cell count, lymph node metastasis assessment [9, 10, 18, 22], breast cancer drug development and others [8], with an accuracy rate of 82.7–92.4% and an AUC score of 0.97 [27, 28]. This also indicated that, with the help of AI, pathological diagnosis and index counting was safe, effective, and feasible [35]. Notably, compared with our IDC identification system, accuracy levels followed the advanced international standards, and this model was a prerequisite to further match the IDC regions with corresponding Ki-67 staining, and to further develop a Ki-67 automatic counting system. However, as far as we know, there are very few such whole-slide-marked ki-67 standards which have accurately labelled each positive and negative cell of ki-67 staining image in public databases, and we will publish these digital Ki-67 images that have been accurately labelled each positive and negative cell by pathologists during the course of this study as an open public databases for other interested researchers.

Factors that lead to poor reproducibility of Ki-67 scoring results may include type of biopsy, time to fixative, type of antibody, method of reading and area of reading [36–39]. To decrease this variability and improve the evaluation of Ki-67, many research institutions including the International Ki-67 Working Group have conducted a series of studies [36–38, 40]. According to the guidelines for the analysis, reporting, and use of Ki-67 proposed by the International Ki-67 in Breast Cancer Working Group, Ki-67 score was defined as the percentage of invasive cancer cells positively stained in the examined region, while staining intensity is not relevant; For type of biopsy, both core-cut biopsies and whole section tissues are suitable, but whole section may give higher Ki-67 scores than core biopsy; For antibody clones, like MIB-1, MM-1, Ki-S5, SP6 and Ventana 30–9, most of the aforementioned studies have been demonstrated that the most widely used and validated antibody is the MIB-1 clone [36–38]. Although some factors like type of biopsy, antibody clones as mentioned above may be correctable, others may be difficult to standardize. The inconsistency in the selection of reading area of slide is generally considered to be one of the important reasons for the poor reproducibility of Ki-67 immunohistochemistry scoring. Due to the heterogeneity of breast cancer, most Ki-67 positive tumour cells are often unevenly distributed, and there are hot spots and cold areas [37, 41]. Many published studies showed that the Ki-67 score obtained by evaluating only the hotspot area or marginal area is significantly higher than the average area, cold area and intermediate proliferation area, and the Ki-67 score in the hotspot area had a greater correlation with breast cancer prognosis [37, 39, 42]. The International Ki-67 Working Group currently recommend that at least three high power fields (HPFs) should be selected to represent the spectrum of staining seen on the initial overview of the entire section, and the invasive edge of the tumour should be counted, and using the average score across the section for the present because of its greater reproducibility [36, 37, 39]. On the other hand, the number of cells counted is also one of the factors affecting the reproducibility of Ki-67 scoring in breast cancer. Obviously, the Ki-67 score obtained by counting 100 tumour cells must be different with 1000 tumour cells on the same immunohistochemistry section. Although there is currently no uniform requirement for the total number of cells in the Ki-67 scoring assessment, many research institutions including the International Ki-67 Working Group have recommend that at least 1000 cells should be scored and that 500 cells be accepted as the absolute minimum to achieve adequate precision [36, 39]. In our present study, Ki-67 was scored by the average method and more than 1000 cells on each Ki-67 slice were counted whether in manual counting or AI stage, which to achieve a harmonized methodology, create greater between-laboratory and between-study comparability of Ki-67 marker in breast cancer.

## Conclusion

Our current study was able to provide computer-based in deep learning by extracting large sample size data information, resulting in the development of automated quantitative analysis of Ki-67 staining and HE images recognition and registration on whole tissue sections in breast carcinoma. We also explored a set of feasible programs and procedures for labelling staff training based on WSI, which further demonstrated that Ki-67 automatic counting system could finish the enumeration with considerably high efficiency and accuracy. In addition, we provide these digital images of Ki-67 staining which have been accurately labelled by pathologists in this study as free-open source. We strongly believe that, with the AI support, pathologists can greatly improve the efficiency and accuracy of Ki-67 counting in breast invasive ductal carcinoma, and efficiently present a more precise and efficient clinical diagnosis. In the near future, we expect to improve more the accuracy and sensitivity of the software by upscaling data and/or algorithms, and then combine it with more immunohistochemical quantitative analysis to develop auxiliary software(s), which could meet the requirements of clinical diagnosis and further pathological applications.

## Supplementary information

**Additional file 1.** Procedures and results of Ki-67 Automatic Counting in breast IDC based on whole tissue sections. The video shows how our automatic counting software can well identify breast IDC regions, and how the identified breast IDC regions (contiguous HE and Ki-67 stained slices) could be properly registered. The software labelled and auto-counted Ki-67 positive and negative tumor cells in respective IDC areas (red for positive cells, green for negative cells) and provided accurate Ki-67 index scores.

## Data Availability

The datasets used during the current study are available from the corresponding author on reasonable request.

## Abbreviations

IDC: Invasive ductal carcinoma
DCIS: Ductal carcinoma in situ
ML: Machine learning
WTS: Whole Tissue Section
WSI: Whole Slide Image
CNN: Convolutional neural networks
HE: Hematoxylin-Eosin
IHC: Immunohistochemical
